# Supplementary material for: OGT Binding Peptide-Tagged Strategy Increases Protein O-GlcNAcylation Level in E. coli
Source: Molecules. 2023 Feb 24;28(5):2129. doi: 10.3390/molecules28052129 (PMC10004047; doi:10.3390/molecules28052129)
Supplement: Supplementary file 1 [file molecules-28-02129-s001.zip › molecules-2165824-supplementary.pdf]

Supplementary Materials

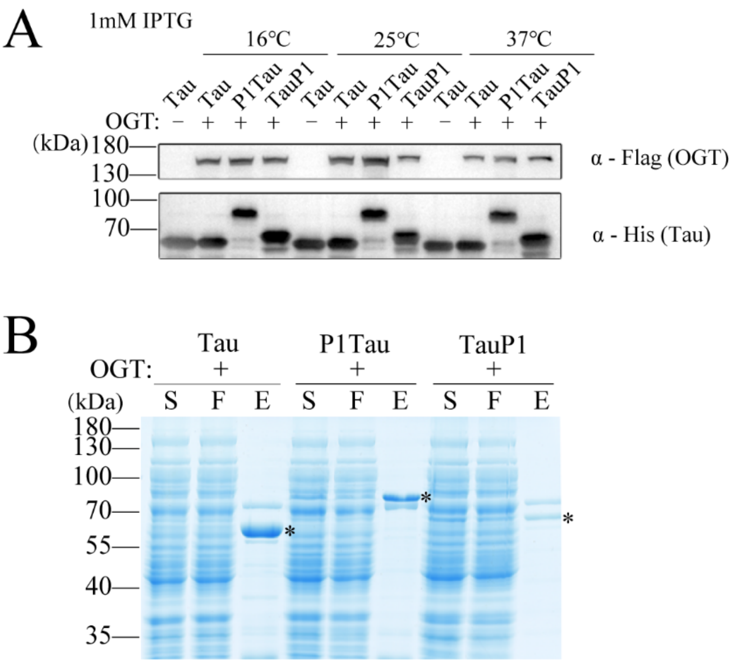

Figure S1. Production of recombinant O-GlcNAc modified Tau and Tagged Tau in *E. coli*. (A) The transformants were grown at 37℃ until OD 600 reached 0.6~0.8. Then the culture was induced with 1 mM IPTG and grown at 16℃, 25℃ or 37℃. The expression of OGT, Tau and tagged Tau proteins were confirmed by WB assay using anti-Flag and anti-His antibody. (B) The O-GlcNAc Tau, P1Tau and TauP1 were purified by Ni-NTA and analyzed by Coomassie brilliant blue staining. S, Supernatant; F, Flow; E, Elution. The location of the asterisk is the target protein.

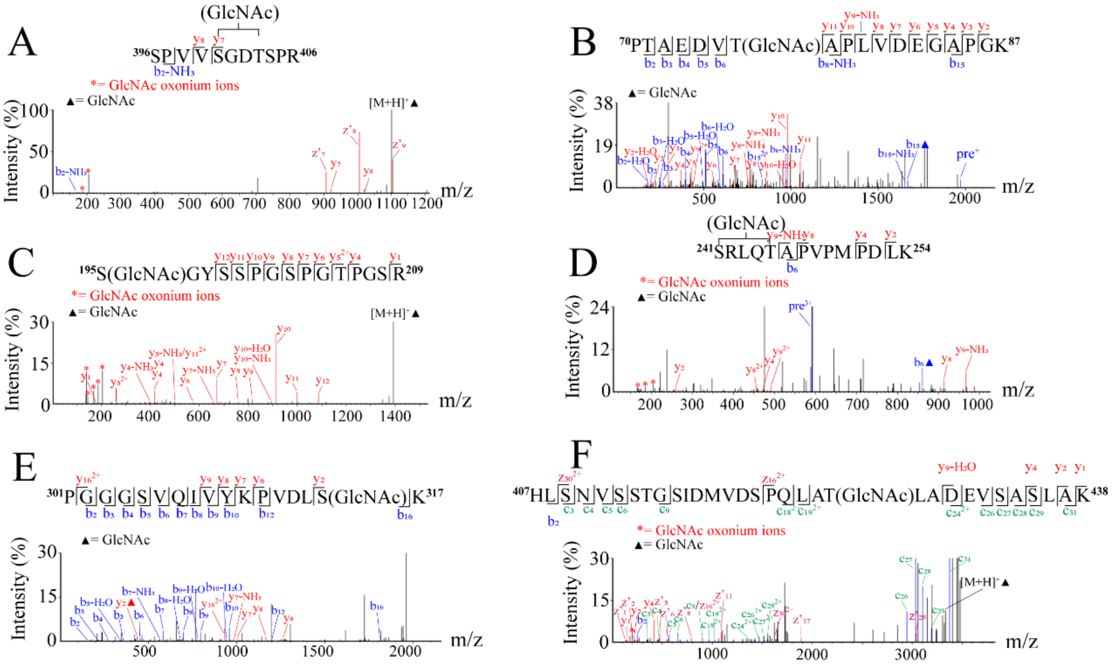

Figure S2. HCD/ETD spectrum reveal other six new O-GlcNAc sites were detected on the following peptides: (A) ^{396}SPVV**S**GD**T**SPR^{406} (P1Tau), S400/T403 indicates O-GlcNAc at one of the sites; (B) ^{70}PTAEDV**T**APLVDEGAPGK^{87} (TauP1); (C)

^{195}**S**GYSSPGSPGTPGSR^{209} (Tau); (D) ^{241}**S**RLQ**T**APVPMPDLK^{254} (Tau), S241/T245 indicates O-GlcNAc at one of the sites; (E) ^{301}PGGGSVQIVYKPVDL**S**K^{317} (Tau); (F) ^{407}HLSNVSSTGSIDMVDSPQLA**T**LADEVSASLAK^{438} (Tau). All of the modification sites shown in underlined letters. Oxonium ions are labeled with red asterisks. Solid triangles represent the presence of GlcNAc modification on the peptide ions and fragment ions.

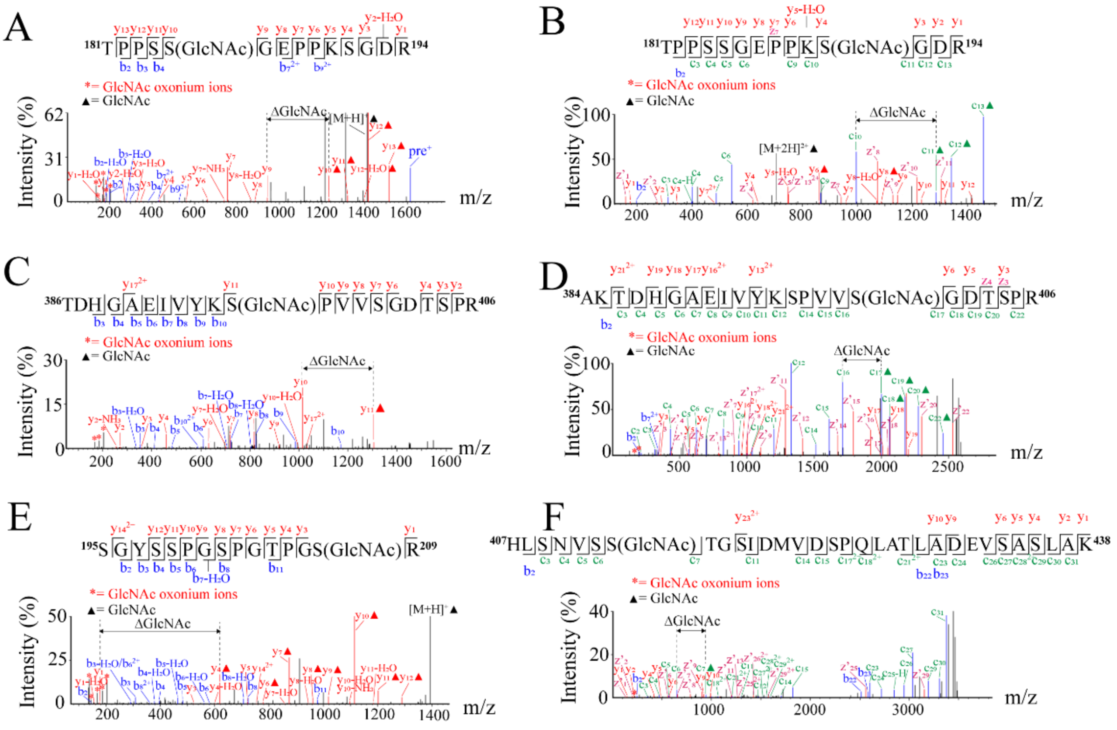

Figure S3. HCD/ETD spectrum reveal some previously reported O-GlcNAc sites were detected on the following peptides: (A) ^{181}TPPS**S**GEPPKSGDR^{194} (TauP1); (B) ^{181}TPPSSGEPPK**S**GDR^{194} (TauP1); (C) ^{386}TDHGAEIVYK**S**PVVSGDTSPR^{406} (P1Tau); (D) ^{384}AKTDHGEIVYKSPVV**S**GDTSPR^{406} (P1Tau); (E) ^{195}SGYSSPGSPGTPG**S**R^{209} (Tau); (F) ^{407}HLSNVS**S**TGSIDMVDSPQLATLADEVSASLAK^{438} (Tau). All of the modification sites shown in underlined letters. Double-headed arrows and ΔGlcNAc indicate the mass shift of the O-GlcNAc moiety between key fragment ions. Oxonium ions are labeled with red asterisks. Solid triangles represent the presence of GlcNAc modification on the peptide ions and fragment ions.
